# The accuracy and retention of presurgical infant orthopaedics constructed from different polymer materials: A comparative study

**DOI:** 10.1016/j.jtumed.2024.01.005

**Published:** 2024-02-05

**Authors:** Raghad Oday, Mushriq Abid, Arkadiusz Dziedzic

**Affiliations:** a Department of Orthodontic, College of Dentistry, University of Baghdad, 01110, Iraq; b Department of Conservative Dentistry with Endodontics, Medical University of Silesia, Katowice, Poland

**Keywords:** جراحة العظام قبل الجراحة, تغذية, بولي ميثيل ميثاكريليت, مصفف شفاف صلب, مصفف شفاف صلب وناعم مزدوج الطبقات, Accuracy, Cleft lip and palate, PET-G/EVA, PMMA, Presurgical orthopaedics

## Abstract

**Objectives:**

This laboratory-based study aimed to evaluate and compare the accuracy and retention of moulding plates when used as pre-surgical orthopaedic appliances (PSIOs) for infants with cleft lip and/or palate (CL/P).

**Methods:**

Ten moulding plates were fabricated from three different materials (total sample size: 30), including polymethyl methacrylate (PMMA), a hard clear aligner (PET-G polymer), and a dual-layered hard and soft clear aligner (mixed PET-G/EVA) on ten three-dimensional (3D) printed working models. Accuracy was evaluated by measuring the virtual gap between the data acquired from the moulding plate and the working model after the optical scanning at each of the designated 36 points for each plate. Exocad software was used to facilitate all virtual alignments and measurements. Retention was measured using a digital gauge that quantified the traction force required to separate the plates from the retention test cast (a soft resin printed cast).

**Results:**

PET-G plates exhibited the best fit with the working cast, with overall adaptations of 0.146 ± 0.012 for PET-G, 0.250 ± 0.073 for PET-G/EVA, and 0.294 ± 0.113 for PMMA. For region-specific misfit, PET-G plates exhibited superior accuracy across all regions, with mean discrepancies of 0.16 ± 0.08 mm, 0.15 ± 0.061 mm, and 0.12 ± 0.128 mm in the anterior, middle, and posterior regions, respectively. Retention for PET-G was significantly higher than the other materials, with a mean of 3.34 N ± 0.487, as opposed to 1.65 N ± 0.331for PMMA and 1.27 N ± 0.239 for PET-G/EVA (P < 0.05).

**Conclusions:**

Moulding plates constructed from PET-G exhibited a better fit and higher retention than those made from PET-G/EVA and PMMA.

**Clinical significance:**

Collectively, our findings suggest that the selection of PET-G for PSIO appliances could have clinical significance by potentially improving treatment outcomes in infants with CL/P.

## الملخص

**أهداف البحث:**

تهدف هذه الدراسة المخلوطة والمختبرية إلى تقييم ومقارنة الدقة والاحتفاظ بألواح التشكيل المستخدمة كأجهزة تقويم العظام قبل الجراحة عند الرضع الذين يعانون من الشفة المشقوقة و/أو الحنك المشقوق.

**طريقة البحث:**

عشرة ألواح قولبة من كل مادة من ثلاث مواد: بولي ميثيل ميثاكريليت، ومصفف شفاف صلب، ومصفف شفاف صلب وناعم مزدوج الطبقات على نماذج عمل مطبوعة ثلاثية الأبعاد. تم تقييم الدقة عن طريق قياس المسافة الافتراضية بين لوحة التشكيل وبيانات المسح الخاصة بطاقم العمل بعد المسح البصري لكليهما عند 36 نقطة لكل لوحة. قام برنامج إكسوكاد بتسهيل جميع المحاذاة والقياسات الافتراضية. قام مقياس رقمي بقياس قوة الجر، التي تمثل قوة الاحتفاظ، باستخدام قالب مطبوع من الراتنج الناعم.

**النتائج:**

أظهرت ألواح المصففات الصلبة والشفافة أفضل ملاءمة مع طاقم العمل، مع تعديلات إجمالية تبلغ 0.146 للمصففات الصلبة الشفافة، و0.250 للمصففات الشفافة الصلبة والناعمة ذات الطبقة المزدوجة، و0.294 للمصففات متعددة الميثيل. بالنسبة إلى عدم التوافق الخاص بالمنطقة، أظهرت لوحات المصففات الصلبة والواضحة دقة فائقة في جميع المناطق، مع متوسط تباين قدره 0.16 مم، 0.15 مم، و0.12 مم في المناطق الأمامية والمتوسطة والخلفية، على التوالي. كان الاحتفاظ ب محاذاة صلبة وواضحة أعلى بكثير مقارنة بالمواد الأخرى، بمتوسط 3.34 ن، مقابل 1.65 ن لميثاكريلات البولي ميثيل و1.27 ن للمصففات الشفافة الصلبة والناعمة ذات الطبقة المزدوجة.

**الاستنتاجات:**

أظهرت ألواح التشكيل المصنوعة من مصففات صلبة وشفافة ملاءمة أفضل واحتفاظ أعلى من تلك المصنوعة من مصففات شفافة صلبة وناعمة مزدوجة الطبقات وميثاكريلات البولي ميثيل. يشير هذا إلى أن المصففات الصلبة والشفافة قد تكون المادة المفضلة لبناء أجهزة تقويم العظام قبل الجراحة للشفة المشقوقة و/أو الحنك المشقوق للرضع.

## Introduction

Cleft lip and/or palate (CL/P) is the most common congenital anomaly affecting the orofacial region with an incidence of 0.94 out of every 1000 live births worldwide.^1^ Patients with CL/P experience difficulties eating and speaking while also experiencing aesthetic and psychological problems; consequently, it is preferable to manage these patients with a multi-disciplinary team of specialists.^2^, ^3^, ^4^ Cleft patients usually undergo numerous surgeries throughout their lives. To reduce the number of surgeries, a variety of techniques and treatment plans have been developed over recent years.^5^

Presurgical infant orthopaedics (PSIO) plays a crucial role in the management of CL/P in infants.^6^^,^^7^ Many PSIO appliances have been reviewed in the literature, including passive, active, and semi-active nasoalveolar appliances.^8^ Most of these moulding plates are made of acrylic resin polymethyl methacrylate (PMMA), which is a most widely used material.^9^ However, previous research reported that acrylic resin may cause oral mucosal irritations, inflammation, and allergic reactions.^9^^,^^10^ Moreover, denture bases manufactured from PMMA are vulnerable to bacterial adhesion and microbial colonization in the oral cavity.^11^^,^^12^ Rougher surfaces are considered a major contributory factor to microbial colonization as these surfaces are difficult to clean and favour regrowth by surviving organisms rather than complete surface re-colonization.^13^

Consequently, traditional PSIO construction methods could impose a significant burden on families.^14^^,^^15^ To overcome these drawbacks, a novel method was developed to fabricate moulding plates which featured the creation of a series of presurgical vacuum-formed PSIO aligners.^9^^,^^16^ This technique was designed to promote the successful movement of alveolar segments and efficient moulding mechanics, to reduce the spent with clinicians, and to reduce the number of treatments and recall visits.^9^^,^^16^^,^^17^

Over recent years, researchers have developed non-invasive, digital techniques to use Computer-aided design (CAD) and/or Computer-Aided Manufacturing (CAM) technologies to engineer PSIO appliances.^18^, ^19^, ^20^ Digitally designed PSIOs offer significant advantages, including enhanced precision in planning the anticipated movement distance and rotational angles of the alveolar segments during each stage of treatment.^17^ Furthermore, this innovative technology allows for optimal mucosal adaptation in the moulding plate, thus leading to improved retention.

Hanno et al. created a digitally designed moulding plate by applying an additive manufacturing process.^20^ In contrast, other researchers employed a subtractive method (milling) for the fabrication of plates.^21^^,^^22^ In another study, Ritschl et al. conducted a comparative study between three-dimensional (3D)-printed moulding appliances and the traditional Grayson technique, and found that these methods were equally effective.^21^

In another study, Shen et al. utilized 3D planning, including alveolar segmentation and the approximation of alveolar segments, along with 3D printing to produce a series of casts. These casts were then used to fabricate plates using conventional acrylic material.^22^ Given that 3D-printed plates are not currently approved by the FDA for intraoral use in infants within the United States, Bous et al. adopted for an alternative method and utilized a clear aligner thermoforming material to fabricate PSIOs on 3D-printed casts. This approach was designed to expedite the manufacturing process while still capitalizing on the advantages offered by CAD systems.^17^

Batra et al. adopted a strategy involving the utilization of clear aligners and 3D planning for presurgical orthopaedic treatment. This approach significantly reduced the burden of care for both infants and parents, and provided a streamlined fabrication process while capitalizing on the benefits offered by CAD.^16^

Furthermore, the utilization of clear aligners streamlines the moulding procedure by eliminating the laboratory procedures traditionally associated with the Grayson technique. This simplification results in a more resource-efficient and convenient process for both the patient and the caregiver, as it reduces the necessity for multiple visits for laboratory adjustments. Clear aligners also provide enhanced visibility of the soft tissues, enabling a more thorough assessment of blanching and sore spots. Overall, these factors enhance the overall comfort and clinical experience of the patients.^13^^,^^18^

The retention of PSIO is important for the appropriate approximation of segments, to prevent choking and injury during feeding, and to minimize the treatment time as frequent dislodgments of the appliance can lead to a prolonged treatment time.^23^, ^24^, ^25^, ^26^, ^27^ In addition to considerations related to safety and material durability, achieving a high level of accuracy is crucial if we are to ensure the optimal retention of PSIO appliances.^24^^,^^28^

To increase the retention of these appliances, some clinicians use adhesive tapes and denture adhesives; however, adhesive tape causes skin irritation, while denture adhesive is difficult to remove from a patient's mouth and can induce microbial growth; furthermore, dental adhesive contains zinc which exerts cytotoxic effects.^29^, ^30^, ^31^, ^32^, ^33^

In this study, our primary goal was to investigate and compare the accuracy and retention properties of PSIO plates fabricated from three different materials: Orthodontic Clear Polymethyl Methacrylate (PMMA), Polyethylene Terephthalate modified with Glycol (PET-G), which is commonly used for hard and clear aligners, and a combination of PET-G and Ethylene Vinyl Acetate (EVA) for dual clear aligners. The null hypothesis posited that there would be no significant difference in the fitness and retention between materials being compared.

## Materials and Methods

### Fabrication of individual orthopaedic appliances

We took an impression for an infant (aged 2 months) with unilateral CLP using a fast-set polyvinylsiloxane impression material (Prestige, Italy) in a special paediatric acrylic tray with the infant fully awake and in an almost upright position; during the procedure, the infant was held by one of the parents. The impression was poured with type IV stone (Zhermack S.p.A., Zhermack, Italy), and the stone model was scanned with an E1 optical extraoral scanner (3Shape TRIOS extraoral scanner Copenhagen, Denmark). The resultant scan data was stored as a standard tessellation language (STL) file and imported into Exocad in-lab Dental CAD. The deep undercut in the area of the cleft was blocked virtually before the models were printed. The materials used in this study are listed in Table 1.

**Table 1:** The materials used in the study.

Item	Origin
Polymethylmethacrylate resin (PMMA)	Orthocryl from Dentaurum GmbH and Co.KG, Ispringen, Germany
Single layers of square plates made of polyethylene terephthalate modified with glycol.	Leone, Italy
Dual layers of polyethylene terephthalate modified with glycol and ethylene vinyl acetate (PET-G/EVA)	Leone, Italy
Fast-set addition polyvinylsiloxane impression material	Prestige, Italy
Type IV stone	Zhermack, Italy
Polygrip zinc-free denture adhesive cream	Polygrip, Haleon- Ireland
Glycerin aqueous solution (80 %)	MP Biomedicals -Santa Ana, California, United States.
Resin for 3D printing dental models, dental gum resin	Asiga, Australia
Titanium dioxide spray	Dentaco, Germany

Digital data were exported to print the study models by stereolithography (SLA); ten models were printed for plate fabrication, and one model was printed with 2 mm soft gum for the retention test (Asiga Dental Model, Asiga Dental Gum Resin, Australia).

Thirty moulding plates were constructed on the printed models, ten from each material; the first material was PMMA (Orthocryl resin-based, Dentarum-Germany), the second was a PET-G clear aligner (PET-G, Leone, Italy), and the third was a PET-G/EVA clear aligner (PET-G, Leone, Italy). Ten moulding plates were fabricated from each material on the printed models, and one plate from each material was fabricated on each model.

All of the materials used for plate construction have been approved by the FDA. A vacuum form machine (Scheu Dental Biostar Thermoforming Unit, Germany) was used to fabricate 2 mm aligners (PET-G, PET-G/EVA). The plates were removed from the model carefully to avoid unnecessary trimming or breakage, and all rough borders or ridges were finished thoroughly with handpiece burs.

The production of conventional moulding plates involved a skilled clinician crafting one plate for each model. Separating Agent (SND, Shanghai New Century Dental Materials Co., Ltd, Shanghai, China) was sprayed onto each model. After a brief drying period, the model was completely dried using compressed air. Subsequently, a cold polymerizing PMMA, consisting of a liquid methyl methacrylate monomer and polymethyl methacrylate powder, was used to fabricate moulding plates using the salt and pepper technique, as recommended by the manufacturer.^33^ The models were then polymerized in a polymerization pressure vessel (Dentarum Polyclav, Ispringen, Germany) for 30 min at 40 °C and 3 bars of pressure.

Subsequently, the plates underwent further post-processing following traditional dental technician procedures, including the removal of excess material, smoothing, and polishing. To achieve the desired finish, the PMMA plates were first treated with acrylic burs to eliminate any acrylic excess. Then, the plates were finished using sandpaper with a grain size of 120 μm, and to prevent overheating during the finishing process, the specimens were regularly immersed in a rubber bowl filled with water for 15 s.^34^

### Data acquisition and measurement

#### The scanning processes

All samples were scanned using an E1 optical extraoral scanner (with an accuracy of 10 μm) which was calibrated before each set of samples were tested. Each individual sample was placed and secured on the scanning platform using modelling clay. The inner side of the palatal area of the samples was positioned in parallel to the camera module, to form the region of interest. To enhance scanning quality, the specimens were treated with an anti-glare spray, and titanium oxide powder from Dentaco (Germany). To optimize the scanning process, this powder was applied twice from different directions. The 3D parameters of the tissue surface of each specimen and their corresponding models were scanned individually and then each plate was placed on its model to be scanned together.

#### Virtual matching processes

**Figure 1:**
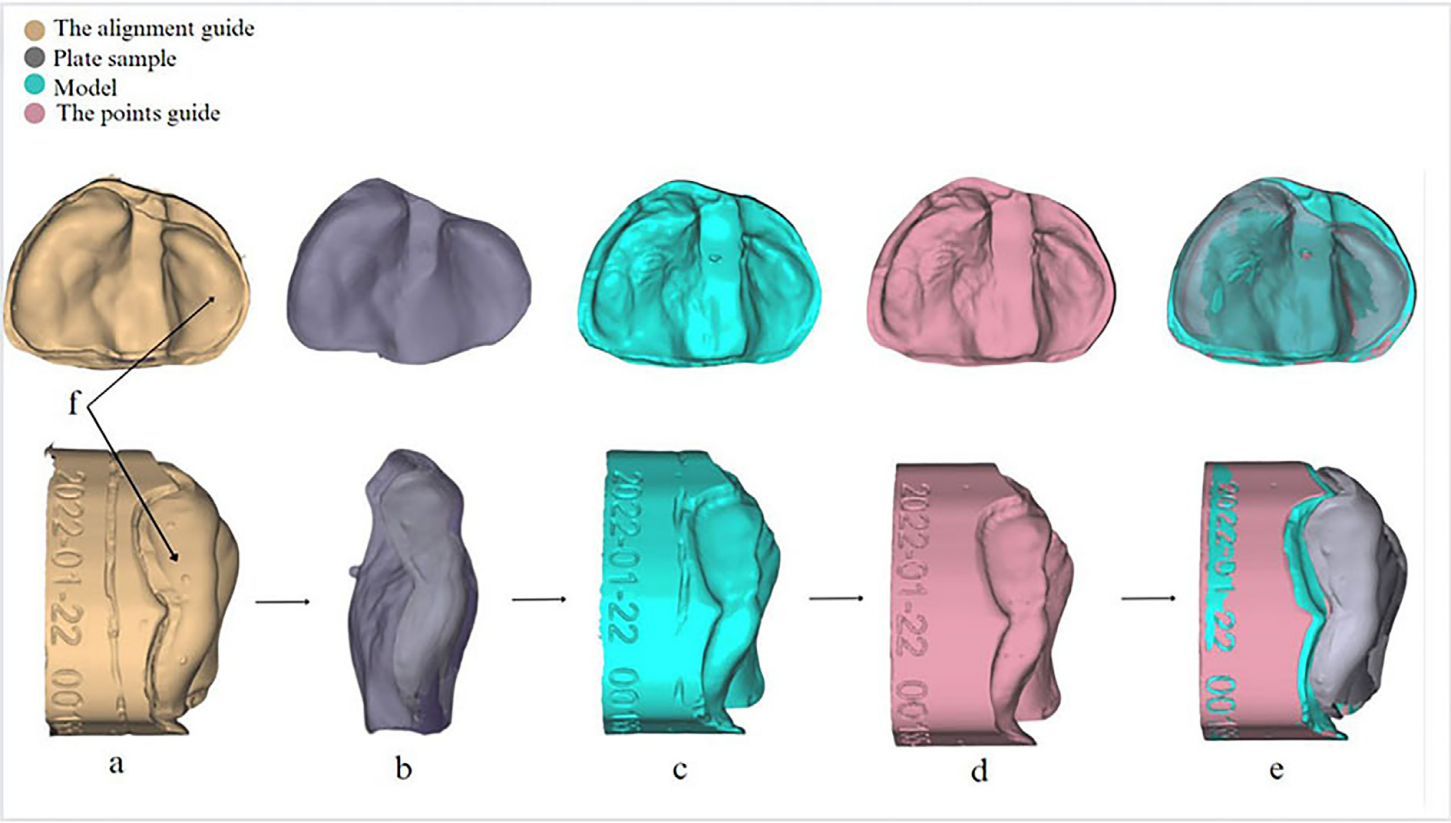
Virtual setup showing the alignment process: **(a)** First, the alignment guide was inserted into Exocad software; **(b)** the plate was inserted and aligned with the alignment guide by considering indentation marks (arrowed); **(c)** the model was inserted and aligned with the alignment guide and the plate; **(d)** the points guide was inserted; and **(e)** finally, the alignment guide was hidden, while the model, plate, and points guide were aligned together for analysis.

Virtual alignments were performed using Exocad software (Exocad Dental 3.1, Darmastadt, Germany). The entire process was based on three scan files (STL files generated from the model, plate, and the model and plate together); these files were imported into Exocad in sequence. Scan data from the plate and the model together were used to act as a template (an alignment guide) on the model and the plate was aligned correctly by referring to indentation marks. Prior to the scanning process, a number of indentation marks were distributed on the model and the plate with a fine handpiece bur to facilitate the virtual alignment of the model and the plate to the alignment guide.^35^ The virtual configuration of the alignment procedure involved a stepwise process in Exocad software. First, the alignment guide was imported. Then, the plate was placed and aligned with the guide by reference to the indentation marks. Subsequently, the model was introduced and aligned with both the alignment guide and the plate. This was followed by integration of the points guide. Finally, the alignment guide was concealed, leaving the model, plate, and points guide precisely aligned and ready for assessment (Figure 1).

#### The measurement process

**Figure 2:**
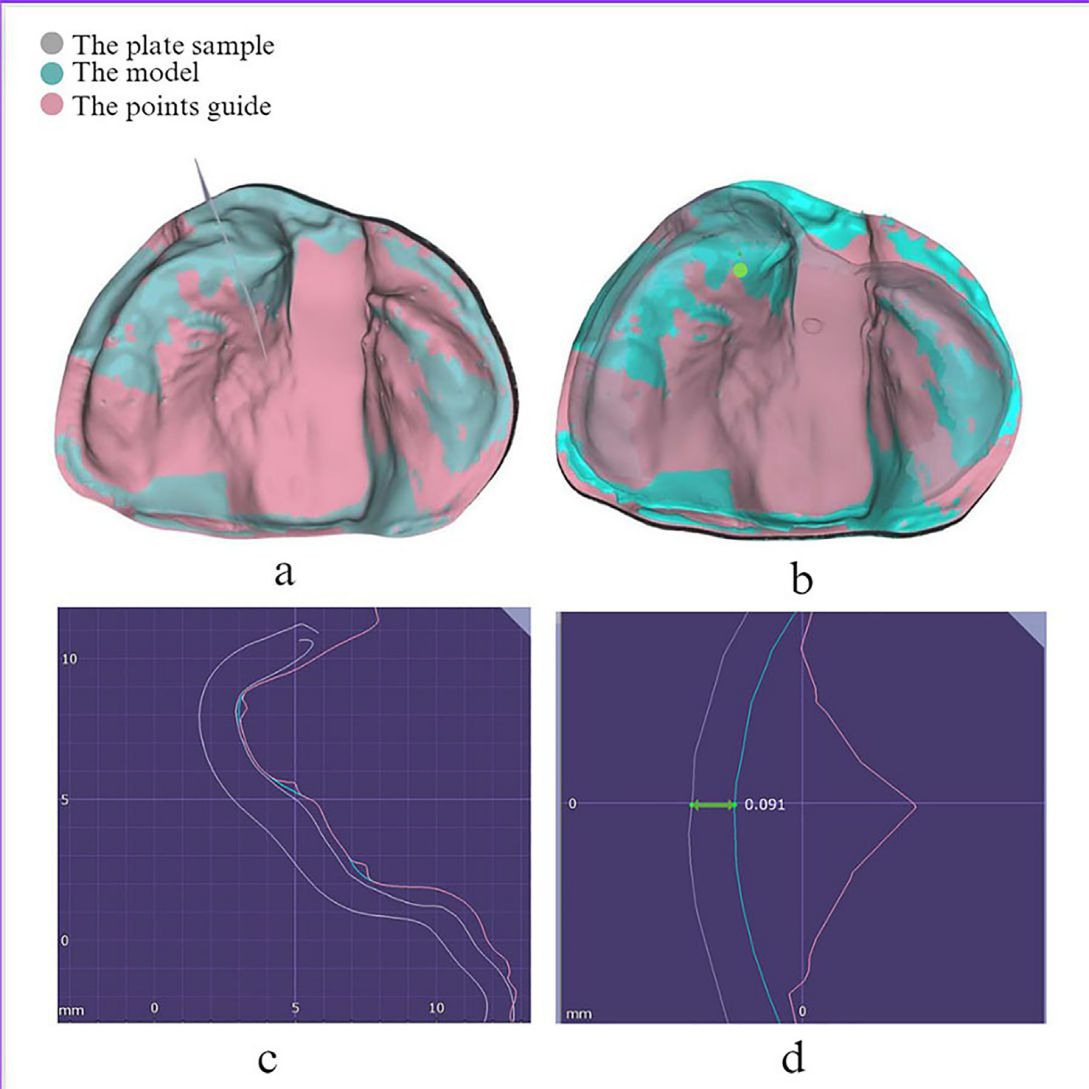
The measurement method used in this study: **(a)** the sector that passed perpendicular through the points (arrowed; **(b)** increased focus on an individual point (arrowed); **(c)** a sector passing through the cast, plate, and the points guide at the selected points; **(d)** the cast and the molding plate lines aligned parallel to each other at the horizontal line where the distance was measured in mm.

The points of measurement were created virtually on the original STL file, thus generating a point's guide which acted as a template to standardize the location of each point between samples and materials; thus, the measurements were taken at the same points for each sample. The points guide consisted of 36 points divided into three groups (anterior, middle, and posterior); there were 12 points for each group (six for the right, and six for the left side). Once the alignment process was complete, we virtually measured the gap or distance between the inner surface of the plates and their corresponding models; this procedure was carried out at identical points for all sample sizes. Figure 2 illustrates a sector passing perpendicularly through the designated points. The measurement process involved focussing on an individual point, where the sector precisely intersected the cast, plate, and points guide at the selected points.

### The measurement of retentive force

**Figure 3:**
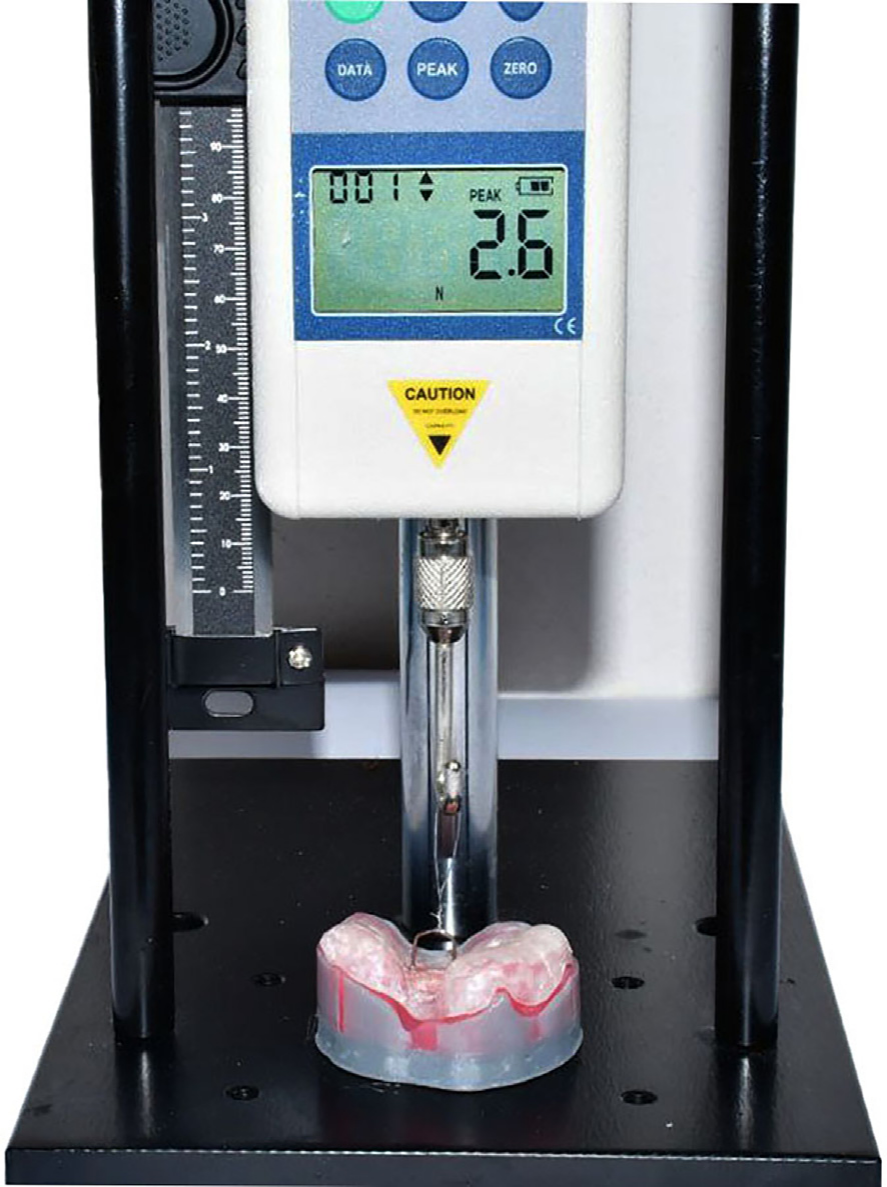
A moulding plate and cast with soft gum were placed on a traction device toinvestigate retention force.

The assessment of PSIO plate retention was conducted using a cast model prepared from soft gum and printed from the same STL file used for the working cast. To measure retention, we used a traction device with a digital force gauge (500 N Mxmoonfree Digital Force Gauge, USA); this device had a resolution of 0.1 N and an accuracy of 0.5 %. The printed soft cast was securely positioned to the stand of the traction device with the palate facing upwards; in addition, we used aqueous 80 % glycerol solution as a lubricant on the cast to simulate the oral cavity (Figure 3).

Polygrip Zinc-free Denture Adhesive Cream (Haleon, Ireland), a standard commercial denture adhesive paste, was specifically applied between the maxillary model and the experimental feeding plate. A plastic syringe was used to ensure the consistent and standardized application of adhesive. Then, we applied a pressure (10–12 Newtons (N)) to the moulding plate for 20 s to ensure an even distribution of adhesive across the entire mucosal surface of the experimental plate, as described previously.^36^ Following pressurization, a traction force was exerted on the experimental sample, commencing from the position that represented the centre of gravity and directed upwards. The traction force was applied directed perpendicular to the imaginary occlusal plane. The point at which the moulding plate separated from the cast was recorded as the retentive value, and represented the maximum traction force. This test was repeated three times for each sample, and the mean value derived from these three tests was recorded as the retentive force.

### Statistical analysis

Data were analysed by SPSS Statistics version 25 (IBM, Armonk NY, USA). Normality and homogeneity were evaluated by applying Shapiro–Wilk's and Levene's tests. Means, standard deviations (SDs), and 95 % confidence intervals (CIs) were calculated. One-way analysis of variance (ANOVA) was performed in conjunction with post hoc analysis to determine if there were statistically significant differences among different regions and materials. Welch test, Tukey's HSD and Games-Howell value were used to test the significant differences between groups. The significance level for statistical tests was α = 0.05; an α = 0.01 presented the highest statistical significance.

### Reliability analysis

Intraclass correlation coefficients (ICCs) were calculated to determine the intra-examiner reliability for accuracy; these calculations involved ten samples which were measured twice separated by a one-week interval. Analysis indicated substantial agreement between measurements under both the ‘Single’ and ‘Average’ conditions (0.995a, 0.998c), thus signifying consistent and reliable ratings or measurements within a tightly defined CI. With regards to the retention test, both the ‘Single’ and ‘Average’ conditions exhibited a moderate to high level of agreement (0.935a, 0.966c) between measurements. Collectively, these data indicate that the measurements acquired from the same subjects were reasonably consistent.

### Ethical considerations

Informed consent was obtained from the parents or legal guardians of all of the children involved in this study. The study was approved by the Ethical Committee of the College of Dentistry, University of XXX (Reference number: 613422; Approval date: 10-4-2022).

## Results

Thirty moulding plates were constructed on the printed models, ten from each material. Ten moulding plates were fabricated from each material on the printed models, and one plate from each material was fabricated on each model.

### PSIO fitting

#### Overall surface adaptation

The distance between the inner surface of the plates and their corresponding models was measured at 36 measuring points. Plates that had been fabricated in a conventional manner with PMMA exhibited a mean ‘fitting’ value of 0.294 ± 0.113 mm (which represent the distance (the gap) between the molding plates and their corresponding models). This compared to 0.250 ± 0.073 mm for the dual clear aligner. Interestingly, the moulding plates constructed from PET-G exhibited the best accuracy and the smallest gap discrepancy (0.146 ± 0.012 mm) (Table 2).

**Table 2:** Descriptive values of accuracy data for the overall area of each type of moulding plates.

Descriptive values	Accuracy		
	PET-G	PET-G/EVA	PMMA
Mean	0.146	0.250	0.294
St. Deviation	0.012	0.073	0.113
St. Error	0.004	0.023	0.036
Minimum	0.128	0.137	0.129
Maximum	0.165	0.381	0.429
Shapiro–Wilk's test value	0.865	0.841	0.210
Levene's test value	<0.001	<0.001	<0.001

PMMA: Polymethyl Methacrylate; PET-G: Polyethylene Terephthalate modified with Glycol; and EVA: Ethylene Vinyl Acetate.

The values shown in Table 1 were normally distributed, but exhibited variance in terms of homogeneity. Consequently, we used Welch's test instead of one-way ANOVA for statistical analysis. No significant differences were identified when comparing between moulding plates fabricated from PMMA and PET-G/EVA (p = 0.241; Games-Howell value). There was a highly significant difference (p < 0.001) when comparing the data derived from PET-G moulds to the PMMA and PET-G/EVA moulds (Table 3).

**Table 3:** Welch and Games-Howell values relating to overall accuracy.

*p* value from Welch's test		<0.001
*p* value (Games-Howell)		
PET-G/EVA	PET-G	<0.001
	PMMA	0.241
PET-G	PMMA	<0.001

**Figure 4:**
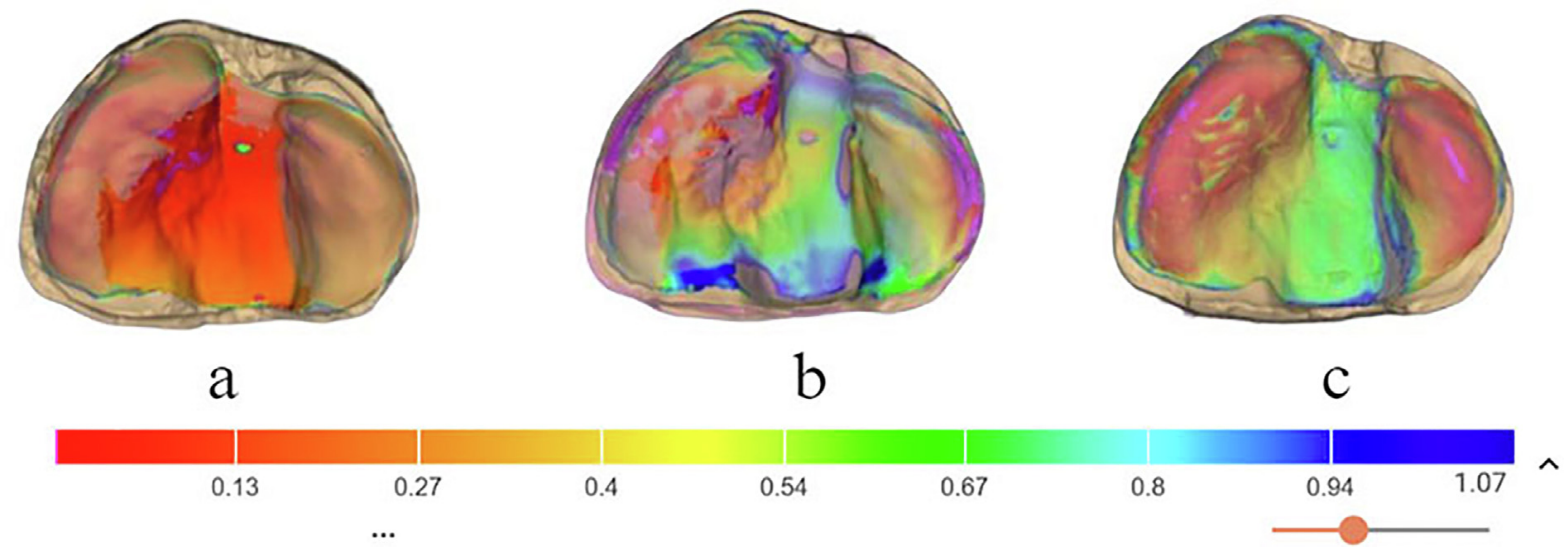
Comparison of the profiles between models and their experimental moulding plate fabricated from **(a)** PET-G, **(b)** PET-G/EVA, and **(c)** PMMA materials. The colour bar represents the distance in mm.

Figure 4 presents a colour map displaying differences in the profiles of the experimental moulding plates fabricated from the three different materials when superimposed with compatible models. The PSIO plates made from each material showed various tendencies considering the range of surface adaptation. The plates generated from PET-G material exhibited constant and consistent adaptation within the ridge areas (shown as red to orange in the scale in Figure 4) with slight pressure areas at the centre of the palate (shown as red in the scale in Figure 4). Conversely, the plates prepared from the dual and PMMA materials exhibited inconsistencies in their adaptation profiles; the presence of purple, green, and blue colours in Figure 4 represents suboptimal adaptation. Specifically, the purple colour represents an inwards deviation, whereas the green and blue colours represent outwards deviation (in mm).

#### Region-specific misfit

For all three of the materials, the greatest misfit was identified in the anterior regions. PET-G moulding plates showed the most optimal fit for all regions when compared to the other materials (Table 4).

**Table 4:** Descriptive values for region-specific accuracy.

Descriptive values	Accuracy								
	PET-G			PET-G/EVA			PMMA		
	Ant.	Mid.	Post.	Ant.	Mid	Post.	Ant.	Mid.	Post.
Mean	0.160	0.151	0.129	0.274	0.233	0.243	0.313	0.278	0.293
St. Deviation	0.080	0.061	0.028	0.085	0.088	0.058	0.063	0.156	0.150
St. Error	0.025	0.019	0.009	0.027	0.028	0.018	0.020	0.050	0.047
Minimum	0.069	0.090	0.081	0.128	0.130	0.152	0.206	0.062	0.084
Maximum	0.270	0.261	0.158	0.405	0.446	0.321	0.388	0.549	0.474
Shapiro-wilk's test value	0.064	0.152	0.147	0.749	0.092	0.576	0.394	0.507	0.212

One-way ANOVA and Welch's test revealed a significant difference between PET-G and the other materials, as shown in Table 5.

**Table 5:** Inferential statistics (Levene's test, ANOVA, and Welch's test) for region-specific accuracy.

Inferential statistics	*p* Value (Levene's value)	*p* Value (ANOVA)	*p* Value (Welch's test)
Ant.	0.561	<0.001	
Mid.	0.014		0.027
**Post.**	<0.001		<0.001

Anteriorly, post-hoc analysis tests for PET-G moulding plates revealed a highly significant difference when compared with the PMMA and PET-G/EVA PSIOs (p < 0.001 with PMMA; p = 0.007 with PET-G/EVA). However, no significant difference was identified between the PMMA and PET-G/EVA plates (p = 0.503). Similarly, no significant difference was identified between the three materials when considering the middle region (Table 6).

**Table 6:** Post-hoc analysis of the anterior, middle, and posterior regions.

Tukey's HSD for anterior regions		*p*
PET-G/EVA	PET-G	0.007
	PMMA	0.503
PET-G	PMMA	<0.001
**Games-Howell value for middle region**		***p***
PET-G/EVA	PET-G	0.071
	PMMA	0.074
PET-G	PMMA	0.083
**Games-Howell value for posterior region**		***p***
PET-G/EVA	PET-G	<0.001
	PMMA	0.608
PET-G	PMMA	0.018

Posteriorly, we identified a highly significant difference when comparing the PET-G and PET-G/EVA plates (p < 0.001) and when comparing the PET-G and PMMA plates (p = 0.018). No significant difference was detected between PMMA and PET-G/EVA when considering the posterior region (p = 0.608); the PET-G/EVA and PMMA plates exhibited the greatest misfit when considering all regions, as shown in Table 6.

### Retention test

Retention testing revealed a highly significant difference (p < 0.001) between the three groups (PMMA, PET-G, and the PET-G/EVA clear aligner). PET-G moulding plates were the most retentive (3.34 ± 0.49 N), followed by PMMA (1.65 ± 0.33 N); the least retentive material was PET-G/EVA (1.27 ± 0.24 N), as shown in Table 7.

**Table 7:** Descriptive values derived from the retention test.

Descriptive values	Retention		
	PET-G	PET-G/EVA	**PMMA**
Mean	3.335	1.270	1.650
St. Deviation	0.487	0.239	0.331
St. Error	0.154	0.076	0.105
Minimum	2.40	1	1.25
Maximum	4.15	1.65	2.25
Shapiro-wilk's value	0.799	0.267	0.465

One-way ANOVA and post-hoc testing (Tukey's HSD) revealed a highly significant difference between PET-G and the two other materials (Table 8).

**Table 8:** ANOVA and Tukey's HSD test data for the retention test.

*p* value (ANOVA)		<0.001
*p* value (Tukey's HSD)		
PET-G/EVA	PET-G	<0.001
	PMMA	0.070

## Discussion

The operational principle of moulding plates shares some similarities with complete dentures, given that both rely on edentulous tissue support. However, it is pertinent to recognize that the fitness of moulding plates can vary due to disparities in the dimensions and proportions of the appliances, and the specific materials employed. One major obstacle that is frequently reported by those who wear removable appliances include insufficient retention and accuracy.^37^^,^^38^ Retention plays a crucial role in the success of orthopaedic therapy, and effective retention is achieved by close adaptation to the mucosal surface.^39^, ^40^, ^41^

The retention of prosthetic removable appliances can be influenced by several physical parameters, including adhesion, cohesion, the thickness of the salivary film, surface tension, and atmospheric pressure.^42^^,^^43^ Undercuts are areas where the denture material can engage with the soft tissue, enhancing stability and retention of the prosthesis within the mouth. If all undercuts are completely blocked out, it can compromise the retention of the appliance, making it more prone to dislodgement during function.^39^ In adults with a complete denture, the posterior palatal seal is situated along the entire peripheral edge of the posterior section of the denture. This serves as a robust barrier and effectively prevents the entry of air or liquids when the soft palate moves during functional activities or when there is slight movement of the denture during function.^40^ Unfortunately, patients with CL/P do not possess a palatal seal; consequently, the effect of atmospheric pressure is weak.^41^

The fitness and retention of PSIO appliances are critical factors in the successful management of infants with CL/P.^24^^,^^41^ A well-fitted and retained PSIO contributes to efficient alignment of the alveolar segment, improves suckling function, and enhances treatment outcomes and patient satisfaction; therefore, it is important to identify materials to manufacture PSIOs in an effective manner without the complications related to acrylic resin or denture adhesive.^24^^,^^25^^,^^27^

Notably, while previous research studies have investigated the precision of various materials in applications, such as splints,^43^ palatal plates,^27^ and dentures,^44^ no previous study has performed a comprehensive evaluation of potential material combinations and technologies (including conventional material, PET-G, and PET-G/EVA) within a standardized testing framework tailored to the specific context of these moulding plates.

In the present study, all of the PET-G plates tested exhibited a significant difference in fitness when compared to PMMA plates; therefore, our null hypothesis should be rejected. Taking into account the entire set of measurements and the manufacturing procedure, it is plausible that the range of the measured values approached the threshold of precision attainable by the scanning device and the associated software. Nevertheless, even within these constraints, it is possible to extract specific and distinctive findings from our results within an academic context.

Our study unequivocally demonstrated that a moulding plate fabricated from PET-G exhibited superior fitness when compared to plates fabricated from PMMA and PET-G/EVA. Conversely, PET-G/EVA plates exhibited the least fitness and retention when compared to plates prepared from the other materials. Only a limited body of evidence is available in the literature relating to the comparison of such accuracy for these materials. Bichu et al. reported that PET-G clear aligners exhibited outstanding transparency, satisfactory flow properties, and a remarkable resistance to various solvents. These aligners are produced by either printing or hot stamping methods. Moreover, PET-G materials are known for their exceptional durability, high impact strength, resistance to chemical alterations, and are the preferred choice for creating intricate and complex designs.^45^

The dimensional stability and precision of moulding plates are pivotal factors that contribute to their close adaptation to oral tissues, ultimately leading to improved fitness and retention. This level of intimate adaptation is critical if we are to ensure the successful performance and retention of an appliance within an infant's mouth.^46^, ^47^, ^48^

In the present study, we found that PET-G/EVA thermoplastic materials exhibited a suboptimal and unacceptable accuracy and retention when compared to the other two materials. This may be attributed to the structure of PET-G/EVA clear aligners which features both PET-G and EVA. The EVA polymer is a semicrystalline, viscoelastic, and flexible material with a glass transition temperature below room temperature; these factors exert impact on the mechanical properties of the material.^49^^,^^50^ Moreover, when fabricating this type of appliance, we observed that less time was needed to prepare the clear aligners when compared to PMMA. Thermoplastic materials provide enhanced visual clarity of the soft tissues, thus enabling a more comprehensive assessment of blanching and sore spots; these effects enhance the overall comfort levels of the patient.^16^

The deviations observed in the colour maps generated by our analysis, along with the lower levels of fitness and retention when the two other materials were compared to the PET-G material could be due to a range of different factors. The manufacturer recommends utilizing the spray-on technique for producing this type of plate^33^; however, the precise ratio of monomer-to-polymer can vary due to the manual process, thus resulting in different levels of shrinkage during polymerization; this can lead to a tighter or looser fit of the appliance.^43^^,^^48^ Such shrinkage may have played a role in the deviations observed in our colour map, as described previously.^43^^,^^51^ Beyond its impact on accuracy, previous research has demonstrated that monomer content can influence a range of factors, including the mechanical properties and the quantity of unreacted residual material.^27^

### Limitations

There are some limitations to this study that need to be considered. For example, this study focussed on complete unilateral CL/P but did not include the clinical use of PSIOs for other types of clefts (cleft palate or bilateral cleft lip and palate). In addition, we did not clinically validate the long-term effectiveness of the moulding plates.

### Clinical implications and further research

The outcomes of our present study have significant clinical implications with regards to the choice of materials for PSIO appliances. We found that individual plates manufactured from PET-G demonstrated satisfactory retention and accuracy, making this material a promising option for such applications in the future. While our findings provide valuable insights into the retention and adaptation of various materials in PSIO appliances, further research is still required. For example, it is important that we perform well-designed and long-term studies to investigate clinical performance, patient comfort, and treatment outcomes associated with these materials; such research would enhance our understanding of the suitability of these materials for PSIO applications.

## Conclusions

Our findings revealed distinct retention properties among the three materials investigated. PET-G aligners demonstrated the highest levels of retention, followed by PMMA-based plates; PET-G/EVA plates exhibited comparatively lower levels of retention. Furthermore, PET-G plates exhibited a superior level of fitness when compared to the other materials, offering precise and accurate adaptation to the mucosa. These findings suggest that PET-G appliances may be more favourable for the manufacture of appliances for presurgical orthopaedic use in infants when considering fitness and retention. In contrast, PMMA-based plates exhibited the least favourable fit characteristics, potentially compromising their efficacy as presurgical orthodontic appliances for infants. Further research is now needed to validate these results and investigate key factors, such as oral microbial flora and biofilm retention on the inner surface of the removable appliances used in pre-surgical orthodontics for infants prepared from PMMA and PET-G/EVA.

## Source of funding

This research received no external funding.

## Conflict of interest

The authors have no conflicts of interest to declare.

## Author contributions

Conceptualization, R.O., and M.A.; methodology, R.O.; validation, R.O, and M.A.; formal analysis, R.O.; investigation. R.O; writing the original draft, R.O, and M.A.; review and editing, the provision of logistic support, M.A, and A.D. All authors have critically reviewed and approved the final draft and are responsible for the content and similarity index of the manuscript.

## References

[1] Mossey P.A., Modell B. (2012). Epidemiology of oral clefts 2012: an international perspective. Cleft Lip Palate Epidemiol. Aetiol. Treat., Front Oral Biol.

[2] Kadhim S.S. (2019). The accuracy of cone-beam computed tomography in the evaluation of boney support of teeth among patients with a unilateral cleft of lip and palate among Iraqi Population. J. Baghdad Coll. Dent..

[3] Ja’far Z.J., Salih B.A. (2015). Congenitally missing and supernumerary teeth among a group of 3 - 12 Years old children with cleft lip and/or palate in Iraq. J. Baghdad Coll. Dent..

[4] Abid M., Al-Groosh D., Dziedzic A., Abed H. (2021). Mothers' knowledge and experience concerning presurgical orthopedic management for infants with cleft lip and palate. J. Orthod. Sci..

[5] Alzain I., Batwa W., Cash A., Murshid Z.A. (2017). Presurgical cleft lip and palate orthopedics: an overview. Clin Cosmet Invest Dent.

[6] McNEIL C.K. (1950). Orthodontic procedures in the treatment of congenital cleft palate. Dent. Rec. (London).

[7] Sabir S., Azhari M., Rokhssi H., Merzouk N., Bentahar O. (2021). Presurgical nasoalveolar molding: an advantageous adjunctive neonatal therapy for cleft lip and palate defects in 2 clinical cases. J. Pediatr. Neonatal Individ..

[8] Abd El-Ghafour M., Aboulhassan M.A., Fayed M.M.S., El-Beialy A.R., Eid F.H.K., Hegab S.E., El-Gendi M., Emara D.M. (2020). Effectiveness of a novel 3D-printed nasoalveolar molding appliance (D-NAM) on improving the maxillary arch dimensions in unilateral cleft lip and palate infants: a randomized controlled trial. Cleft Palate-Craniofacial J.

[9] Al Khateeb K.A., Fotouh M.A., Abdelsayed F., Fahim F. (2021). Short-term efficacy of presurgical vacuum formed nasoalveolar molding aligners on nose, lip, and maxillary arch morphology in infants with unilateral cleft lip and palate: a prospective clinical trial. Cleft Palate-Craniofacial J.

[10] Al-Otaibi1 H.N., Alshaalan N.S., Alqarni R., AlMutairi R.M., Altaweel1 S.M., Alshehri H.A., Alfouzan A.F. (2021). Monomer leakage behavior of conventional and CAD/CAM denture acrylic materials under different pH values. Biosc.Biotech.Res..

[11] Larijani M., Zareshahrabadi Z., Alhavaz A., Hajipour R., Ranjbaran A., Giti R., Soltankarimi V., Zomorodian K. (2022). Evaluation of Candida albicans biofilm formation on conventional and computer-aided-design/computer-aided manufacturing (CAD/CAM) denture base materials. Curr Med Mycol.

[12] Budală D.G., Nicolae Bosînceanu D., Surlari Z., Ioan Virvescu D., Baciu R., Cosmin Bida F., Balcoș C., Țănculescu O., Bosînceanu D.N. (2020). Denture base resins and microbial adhesion-current trends. Rom. J. Med. Dent. Educ..

[13] Giti R., Dabiri S., Motamedifar M., Derafshi R. (2021). Surface roughness, plaque accumulation, and cytotoxicity of provisional restorative materials fabricated by different methods. PLoS One.

[14] Dean R.A., Wainwright D.J., Doringo I.L., Teichgraeber J.F., Greives M.R. (2019). Assessing burden of care in the patient with cleft lip and palate: factors influencing completion and noncompletion of nasoalveolar molding. Cleft Palate-Craniofacial J.

[15] Grill F.D., Ritschl L.M., Dikel H., Rau A., Roth M., Eblenkamp M., Wolff K.D., Loeffelbein D.J., Bauer F.X. (2018). Facilitating CAD/CAM nasoalveolar molding therapy with a novel click-in system for nasal stents ensuring a quick and user-friendly chairside nasal stent exchange. Sci Rep.

[16] Batra P., Gribel B.F., Abhinav B.A., Arora A., Raghavan S. (2020). OrthoAligner ‘NAM’: A case series of presurgical infant orthopedics (PSIO) using clear aligners. Cleft Palate-Craniofacial J.

[17] Bous R.M., Kochenour N., Valiathan M. (2020). A novel method for fabricating nasoalveolar molding appliances for infants with cleft lip and palate using 3-dimensional workflow and clear aligners. Am J Orthod Dentofacial Orthop.

[18] Grill F.D., Ritschl L.M., Bauer F.X., Rau A., Gau D., Roth M., Eblenkamp M., Wolff K., Loeffelbein D. (2018). A semi-automated virtual workflow solution for the design and production of intraoral molding plates using additive manufacturing: the first clinical results of a pilot-study. Sci Rep.

[19] Gong X., Dang R., Xu T., Yu Q., Zheng J. (2020). Full digital workflow of nasoalveolar molding treatment in infants with cleft lip and palate. J Craniofac Surg.

[20] Hanno K., Al Shimy A., Saad M., Habib A., El-Fahham I. (2020). Effect of cad/cam nasoalveolar molding appliance on correction of the nasal deformity in complete bilateral cleft lip and palate. Alexandria Dent. J..

[21] Ritschl L.M., Rau A., Güll F.D., Dibora B., Wolff K.D., Schönberger M., Bauer F.X., Wintermantel E., Loeffelbein D.J. (2016). Pitfalls and solutions in virtual design of nasoalveolar molding plates by using CAD/CAM technology - a preliminary clinical study. J Cranio-Maxillofacial Surg.

[22] Shen C., Yao C.A., Magee W., Chai G., Zhang Y. (2015). Presurgical nasoalveolar molding for cleft lip and palate: the application of digitally designed molds. Plast Reconstr Surg.

[23] Grayson B.H., Santiago P.E., Brecht L.E., Cutting C.B. (1999). Presurgical nasoalveolar molding in infants with cleft lip and palate. Cleft Palate-Craniofacial J.

[24] Levy-Bercowski D., Abreu A., DeLeon E., Looney S., Stockstill J., Weiler M., Santiago P.E. (2009). Complications and solutions in presurgical nasoalveolar molding therapy. Cleft Palate-Craniofacial J.

[25] Dinh T.T.N., Van Nguyen D., Dien V.H.A., Dong T.K. (2021). Effectiveness of presurgical nasoalveolar molding appliance in infants with complete unilateral cleft lip and palate. Cleft Palate-Craniofacial J.

[26] Hotz M.M., Gnoinski W.M. (1979). Effects of early maxillary orthopaedics in coordination with delayed surgery for cleft lip and palate. J Maxillofac Surg.

[27] Aretxabaleta M., Unkovskiy A., Koos B., Spintzyk S., Xepapadeas A.B. (2021). Accuracy evaluation of additively and subtractively fabricated palatal plate orthodontic appliances for newborns and infants-an in vitro study. Materials (Basel).

[28] Khimani F., Livengood R., Esan O., Vos J.A., Abhyankar V., Gutmann L., Tse W. (2013). Pancytopenia related to dental adhesive in a young patient. Am. J. StemCells..

[29] Nunes É.M., Policastro V.B., Scavassin P.M., Leite A.R., Mendoza Marin D.O., Giro G., de Oliveira Júnior N.M., Compagnoni M.A., Pero A.C. (2016). Crossover clinical trial of different methods of removing a denture adhesive and the influence on the oral microbiota. J Prosthet Dent.

[30] Bhuskute A.A., Tollefson T.T. (2016). Cleft lip repair, nasoalveolar molding, and primary cleft rhinoplasty. Facial Plast. Surg. Clin. North Am..

[31] Yamada M., Takase K., Suehiro F., Nishimura M., Murata H. (2020). Effects of denture adhesives and mouth moisturizers to human oral fibroblast and human keratinocyte cells using direct and indirect cell culture systems. Dent Mater J.

[32] López-García S., Pecci-Lloret M.P., García-Bernal D., Guerrero-Gironés J., Pecci-Lloret M.R., Rodríguez-Lozano F.J. (2021). Are denture adhesives safe for oral cells?. J Prosthodont.

[33] Kehlbacher S. (2015). Orthocryl LC of for your appliances.

[34] Hassan A.F.A., Jameel A., Nahidh M., Hamid D. (2019). Evaluation of some mechanical and physical properties of different types of injectable polymer materials used as a base for removable orthodontic appliances. J. Stomatol..

[35] Oday R., Abid M. (Oct. 2023). Accuracy and retention of molding plates used for infants with cleft lip and palate fabricated from different materials: a cross-sectional clinical study. Cleft Palate-Craniofacial J.

[36] Tasaka A., Matsunaga S., Odaka K., Ishizaki K., Ueda T., Abe S., Yoshinari M., Yamashita S., Sakurai K. (2019). Accuracy and retention of denture base fabricated by heat curing and additive manufacturing. J. Prosthodont. Res..

[37] Darvell B.W., Clark R.K.F. (2000). The physical mechanisms of complete denture retention. Br Dent J.

[38] Abdulkareem H.S., Salem S.A. (2020). Comparison between retention of maxillary acrylic and nylon denture base materials. Polytech. J..

[39] Nimonkar S.V., Belkhode V.M., Asiri A.M., Aldossary M.F., Nimonkar P.V. (2021). A method of hollowing the obturator prosthesis and an overview on the pros and cons of the various materials used for hollowing. J. Med. Life.

[40] Chandu G.S., Hema B.S., Mahajan H., Azad A., Sharma I., Azad A. (2014). A comparative study of retention of complete denture base with different types of posterior patal seals - an in vivo study. Clin Cosmet Invest Dent.

[41] Shah R.J., Katyayan M.K., Katyayan P.A., Chauhan V. (2014). Prosthetic rehabilitation of acquired maxillary defects secondary to mucormycosis: clinical cases. J Contemp Dent Pract.

[42] Aretxabaleta M., Xepapadeas A.B., Poets C.F., Koos B., Spintzyk S. (2021). Comparison of additive and subtractive CAD/CAM materials for their potential use as Tübingen Palatal Plate: an in-vitro study on flexural strength. Addit Manuf.

[43] Unkovskiy A., Schmidt F., Beuer F., Li P., Spintzyk S., Fernandez P.K. (2021). Stereolithography vs. Direct light processing for rapid manufacturing of complete denture bases: an in vitro accuracy analysis. J Clin Med.

[44] Marcel R., Reinhard H., Andreas K. (2020). Accuracy of CAD/CAM-fabricated bite splints: milling vs 3D printing. Clin Oral Invest.

[45] Bichu Y.M., Alwafi A., Liu X., Andrews J., Ludwig B., Bichu A.Y. (2022). Advances in orthodontic clear aligner materials. Bioact Mater.

[46] Kumar V.H.C., Surapaneni H., Ravikiran V., Chandra B.S., Balusu S., Reddy V.N. (2016). Retention of denture bases fabricated by three different processing techniques - an in vivo study. J. Int. Soc. Prev. Community.

[47] Hwang H.J., Lee S.J., Park E.J., Yoon H.I. (2019). Assessment of the trueness and tissue surface adaptation of CAD-CAM maxillary denture bases manufactured using digital light processing. J Prosthet Dent.

[48] Ryokawa H., Miyazaki Y., Fujishima A., Miyazaki T., Maki K. (2006). The mechanical properties of dental thermoplastic materials in a simulated intraoral environment. Orthod Waves.

[49] Dias R.B., Coto N.P., Batalha G.F., Driemeier L. (2018). Systematic study of ethylene-vinyl acetate (EVA) in the manufacturing of protector devices for the orofacial system. Biomater. Regen. Med..

[50] Aretxabaleta M., Xepapadeas A.B., Poets C.F., Koos B., Spintzyk S. (2021). Fracture load of an orthodontic appliance for robin sequence treatment in a digital workflow. Materials (Basel).

[51] Einarsdottir E.R., Geminiani A., Chochlidakis K., Feng C., Tsigarida A., Ercoli C. (2020). Dimensional stability of double-processed complete denture bases fabricated with compression molding, injection molding, and CAD-CAM subtraction milling. J Prosthet Dent.

